# New Methods of Treatment Efficacy Research: Bridging Clinical Research and Clinical Practice

**Published:** 1997

**Authors:** Kathleen M. Carroll

**Affiliations:** Kathleen M. Carroll, Ph.D., is an associate professor and scientific director of the Psychotherapy Development Center of the Division of Substance Abuse, Department of Psychiatry, Yale University School of Medicine, New Haven, Connecticut

**Keywords:** addiction care, AODU (alcohol and other drug use) treatment method, research and evaluation method, treatment research, treatment outcome, diagnostic criteria, education and training, health care quality control, treatment follow-up, patient assessment, patient care compliance, procedural manual, managed care, drug therapy, psychotherapy, applied research, literature review

## Abstract

A variety of methodological advances are allowing alcoholism treatment researchers to develop effective new treatments and to answer more complex questions regarding the efficacy of treatments for alcohol use disorders. These advances include the use of standardized diagnostic criteria; thorough description of the study populations; standardization of treatments (e.g., through the use of manuals); training of treatment providers; quality control procedures and manipulation checks; and multidimensional, longitudinal assessments. Many of these methods can be adopted by clinicians to improve clinical care as well as to meet the challenges posed by managed care and other changes in the years ahead.

Research on the efficacy^1^ of treatments for alcohol use disorders (AUD’s) has become considerably more rigorous in recent years, and methodological features that were rarely seen in clinical trials as recently as 10 years ago have now become standard practice (Miller et al. 1995; Morley et al. 1996). The increasing sophistication of treatment outcome research has enabled researchers to investigate numerous relevant and increasingly complex issues. These issues include the effectiveness of different AUD treatments, the relative costs of these treatments, the types of patients who respond to treatment, the symptoms and problem areas that respond to specific treatments, and the long-term benefits of treatment. Moreover, the improvements in treatment outcome research have led to some general conclusions regarding the effectiveness of AUD treatment, as follows (see McCrady and Langenbucher 1996; O’Brien and McLellan 1996; O’Brien 1997):

- Alcoholism treatment generally results in reduced drinking and more efficient use of health care resources.
- Several specific treatments have demonstrated effectiveness.
- Some treatments exhibit differential effectiveness with different patient groups.
- The effectiveness of certain treatment approaches for alcohol and other drug (AOD) use disorders is comparable to that for other chronic disorders, such as diabetes or asthma.
- Each dollar spent on the treatment of AOD use disorders saves between $4 and $12 in long-term societal, economic, and medical costs.

The enhanced methodological rigor in treatment outcome research also has prepared the alcoholism treatment community to meet many of the new challenges generated by the increased presence of managed care. For example, in a managed care environment treatment providers must demonstrate the effectiveness, cost-effectiveness, and value (i.e., factors such as program attractiveness to the patients and patient satisfaction) of the services they deliver (Wexler 1993). Even treatments that are widely used and considered “standard” may continue to be reimbursed only if their effectiveness has been demonstrated empirically. These pressures have already begun to reshape the alcoholism treatment system. For example, long-term inpatient treatment programs have not yet demonstrated superior effectiveness for many patients compared with less expensive outpatient alternatives. As a result, both the length of stay and reimbursements for inpatient programs have been reduced dramatically, even though such programs may be necessary and effective for some patient subgroups (e.g., AOD abusers with severe mental illness). The fact that some comparatively intensive treatments have demonstrated their effectiveness and cost-effectiveness with challenging patient populations (e.g., Linehan et al. 1993), and therefore have continued to be reimbursed, underlines the critical importance of evaluating more intensive treatment models.

This article describes some of the strategies that researchers are using to evaluate the efficacy of various AUD treatment approaches. It also emphasizes “cutting edge” research practices and suggests approaches that might be adopted in clinical treatment programs to help the field meet the challenges ahead. In addition, the article also addresses some of the challenges associated with the application of these methods.

## Methodological Advances in Treatment Research

Many of the newly adopted methodological features in alcoholism treatment research share an emphasis on specificity and standardization of treatment. Thus, virtually all aspects of the treatments evaluated (e.g., how they are implemented, how their effectiveness is assessed, and to whom they are delivered) must be described in detail and specified according to an array of scientific conventions. This strategy has several advantages. First, clear and thorough descriptions make it easier to replicate studies and interventions in different settings. Second, common procedures and assessment instruments facilitate the comparison of results across studies. Third, standardization improves the control of “noise” (i.e., error variance) in the study, thereby enhancing the internal validity of research studies, ruling out alternative explanations of findings, and increasing confidence in the conclusions drawn. Finally, standardization itself may increase treatment efficacy by enhancing the integrity of the treatment and the focus or skill of the therapist (Russell and Orlinsky 1996). This effect, however, has yet to be demonstrated empirically.

The following sections review specific strategies to enhance AUD treatment outcome research by standardizing the characterization of the subject sample, treatment, treatment implementation, and assessment of treatment outcome.

### Strategies To Improve the Characterization of the Subject Sample Standardized Diagnostic Criteria

Many problems that beset early AUD treatment research were associated with variable and imprecise characterization of subjects participating in clinical trials (DeRubeis and Crits-Christoph 1998). For example, the terminology used to describe subjects (e.g., “alcoholics,” “drinkers,” or “individuals admitted for alcoholism treatment”) often differed among studies. Consequently, the samples often varied widely in their severity of alcohol use and comorbid problems. This lack of precision led to enormous difficulties in generalizing from one study sample to another.

The development of standardized diagnostic criteria for AUD’s (e.g., in the American Psychiatric Association’s *Diagnostic and Statistical Manual of Mental Disorders* [DSM] and the World Health Organization’s *International Classification of Diseases* [ICD]) has greatly facilitated the adoption of a common definition of syndromes characterized by lack of control over alcohol use. Equally helpful was the adoption of standardized diagnostic interviews, such as the Structured Clinical Interview for DSM–III–R (SCID) and the Diagnostic Interview Schedule (DIS). Although the definitions of AUD’s continue to evolve (see McCrady and Langenbucher 1996), they provide researchers with standard delineations and thus with a common target to which interventions can be directed.

Current research standards dictate that except for interventions specifically targeting lower-severity alcohol-related problems, people entering a treatment study must meet the DSM–IV (*Diagnostic and Statistical Manual of Mental Disorders, Fourth Edition*) or ICD–10 (*International Classification of Diseases, Tenth Edition*) criteria for current alcohol abuse or dependence. This requirement ensures that only persons with clinically significant AUD’s are included in a trial and justifies their exposure to the potential risks of the treatments studied. This prerequisite is particularly relevant for studies of pharmacotherapies and psychotherapies of unknown efficacy.

The use of standardized diagnostic criteria also facilitates characterization of a sample in terms of comorbid psychiatric disorders that may complicate treatment and affect outcome. For example, variable outcomes may be explained by high rates of psychiatric comorbidity in a sample. Finally, standardized diagnostic criteria allow researchers to describe treatment outcome in terms of clinically significant changes, such as the proportion of subjects whose alcohol dependence is in remission and who no longer meet criteria for current alcohol dependence.

#### Description of the Sample

Morley and colleagues (1996) have stressed the importance of comprehensively describing the study sample in reports of clinical trials. These descriptions should include eight basic characteristics: gender, age, education, marital status, employment, race, number of years of problem drinking, and diagnostic status. A thorough description allows the readers of research reports to determine to what extent a given study sample represents other populations of people with AUD’s. However, surprisingly few clinical trials provide adequate descriptions of their study samples (Morley et al. 1996).

Furthermore, the screening and evaluation process used in a clinical trial should be described in detail. This description should provide the following information:

- How many people comprised the pool from which the subjects were recruited?
- How many people refused to participate?
- How many people did not meet the inclusion and exclusion criteria of the study?
- How many people were randomized to treatment?
- How many people actually started treatment?

Such data, which are reported in only 50 percent of recent AUD treatment studies (Morley et al. 1996), would allow readers to determine how representative the sample is, how attractive the offered treatments are, and how restrictive the inclusion and exclusion criteria used are. For example, in a large study evaluating the efficacy of disulfiram treatment among male veterans (Fuller et al. 1986), 6,629 potential subjects were screened for the study. Only 1,618 of these men met the study’s inclusion and exclusion criteria, and of those, 1,006 did not agree to participate. Only 605 patients ultimately were randomized to treatment, reflecting less than 10 percent of the potential subject pool. Including the results from only a small proportion of all potential subjects, however, may further limit a study’s generalizability (beyond the limits imposed by the study’s inclusion and exclusion criteria) because these results reflect the outcomes of only a select sample.

### Strategies To Improve the Characterization of Treatments

#### Use of Treatment Manuals To Guide Psychotherapy

Treatment manuals specify and describe psychotherapies and provide guidelines to therapists for implementing these treatments. The use of manuals has revolutionized the way clinical research in general is conducted (Luborsky and DeRubeis 1984) and has become a virtual requirement in clinical research of AOD use disorders (Carroll et al. 1994; Morley et al. 1996). Psychotherapy manuals define the theoretical underpinnings, goals, and differences among treatments. In addition, manuals describe the strategies the therapist uses to reach the treatment goals and articulate guidelines that direct the therapist through the treatment process. Finally, treatment manuals also define behaviors prescribed and proscribed for the therapist while conducting treatment.

Psychotherapy manuals serve many purposes, including the following (Kazdin 1995; Luborsky and DeRubeis 1984; Moras 1993):

- Provide a means for objective comparisons of different psychotherapies
- Set standards for the training and evaluation of therapists
- Establish clear treatment goals and clinical care standards
- Foster replication of clinical trials in other settings
- Facilitate the transfer of promising treatments from research to clinical settings
- Reduce clinician allegiance with single treatments by facilitating the clinician’s familiarity with and training in alternative approaches
- Provide a means for identifying effective components of particular treatments and link treatment processes to outcome
- Reduce variability in treatment delivery.

In clinical research, treatment manuals also provide a means of promoting the integrity of treatment over the course of its delivery (e.g., by specifying the essential aspects of a treatment) and of differentiating a specific treatment from other approaches.

Psychotherapy manuals have been criticized as constricting and overly formulaic because they are perceived as emphasizing technique over clinical judgment and skill. For example, psychiatric comorbidity is high among patients in alcoholism treatment, the patients’ motivation for change is variable, and few patients present with simple or single psychosocial problems. Consequently, it is difficult to conceive of a single treatment manual that can provide for appropriate treatment of such a highly heterogeneous patient group.

Although manuals do have limitations, many of those that have been developed for AUD’s tend to be flexible and responsive to clinical needs. Furthermore, manuals do not necessarily restrict clinician judgment or decisionmaking (Wilson 1996). In fact, a focused, empirically based approach to treatment, such as that provided by many manuals, may produce better outcomes than approaches based on clinical judgment, which varies significantly among treatment providers.

Another concern that has been raised regarding the use of manuals is that managed care organizations may prematurely pressure unqualified staff to adopt strict adherence to treatment manuals (Addis and Carpenter 1997). To deliver a manual-guided treatment approach effectively, clinicians may require considerable training and supervision. Because many treatment manuals have been developed for use by experienced therapists, adaptation of manuals by undertrained or unqualified clinicians may result in poorer outcomes that are not comparable to those found in clinical trials. More research is needed on the best strategies for use of treatment manuals in “real world” settings, including studies on the amount and type of training needed for different types of clinicians as well as the degree to which manuals may require adaptation for different clinical settings. However, the good outcomes associated with the three manual-based treatment approaches in Project MATCH (Project MATCH Research Group 1997) underline the flexibility and effectiveness of manual-guided therapies for the highly heterogeneous groups of people with AUD’s.

Despite their current limitations, manuals can help researchers better understand the complex relationships between treatment components and outcome as well as bring information about validated treatments to a more widespread audience. For example, demand for the three psychotherapy manuals developed for the National Institute on Alcohol Abuse and Alcoholism’s Project MATCH has been high, and to date these manuals have been distributed to more than 30,000 clinicians and researchers.

#### Standardization of Treatment in Pharmacologic Trials

In recent years, several effective new pharmacotherapies for AUD’s have been developed, including treatment with naltrexone and acamprosate,^2^ both of which reduce alcohol use (O’Brien 1997). These new pharmacologic agents have been evaluated in randomized clinical trials that meet the rigorous methodological requirements for the approval of new medications by the Food and Drug Administration. The design elements of these trials have included the following:

- Random assignment of patients to treatment
- Double-blind procedures in which neither the subjects nor the investigators know which patients receive the medication
- Use of an inactive substance (i.e., placebo) as a control
- Specification of the formulation and dosage of the medication
- Careful monitoring of patient compliance by monitoring serum levels of the medication or adding to the medication a substance called riboflavin that can be monitored in the patient’s urine (an alternative approach is the use of medication bottles with “medication events monitoring system” [MEMS] caps, which contain a microchip that records the date and time that the bottle was opened)
- Multiple measures of outcome, including patient self-reports, collateral reports, and biological indicators of alcohol use.

A significant problem of many clinical pharmacotherapy studies for AOD use disorders, however, is their inability to retain subjects throughout the course of the study. In most studies of alcohol dependence and other psychiatric disorders, up to 75 percent of the patients drop out of treatment, and of the remaining patients, fewer than one-half are fully compliant with the study medication (Carroll 1997*a*). These high rates of attrition or noncompliance can compromise a study’s validity and its ability to detect statistically significant medication effects. To address this problem, standardization of the psychotherapeutic context in which medications are administered is increasingly important as a design element of pharmacotherapy studies (Carroll 1997*b*), one that links clinical research on psychotherapies and pharmacotherapies.

The goals of standardizing psychotherapy in pharmacotherapy trials include supporting the patient, fostering greater adherence to the treatment protocol, and improving retention and outcome. Research repeatedly has shown that the addition of psychotherapy to pharmacologic trials increases patient retention and compliance with treatment (Onken et al. 1995). Moreover, because psychosocial treatment remains standard care for AUD’s, it is both virtually impossible and unethical to conduct clinical trials in which medications are delivered without some psychosocial support. This is especially true in placebo-controlled studies or in studies evaluating a medication of unknown efficacy. Psychosocial strategies that most often are used to enhance patient retention and compliance include providing support, educating the patient about the disorder and its treatment, monitoring compliance, and offering incentives for retention or compliance (Carroll 1997*b*).

It is also essential that the study’s psychosocial context be controlled and delivered consistently for all patients. For example, if the psychosocial treatment component were allowed to vary, study subjects who were doing poorly (e.g., because they were receiving the placebo and not the medication) might receive more psychosocial therapy and support from study staff. These variations could undermine the likelihood of detecting a treatment effect. Uncontrolled variability in the psychosocial study component also can undermine the study’s statistical power to detect differences between treatments. Thus, specifying and controlling the psychosocial context in which medications are delivered in pharmacotherapy trials is an important strategy for decreasing uncontrolled variability, preventing attrition and noncompliance, addressing ethical concerns, and enhancing the statistical power of clinical trials (Carroll 1997*b*).

### Measures To Improve Treatment Implementation

#### Provider Training

Although psychotherapy manuals offer numerous advantages in clinical and research settings, the treatments they specify can, in practice, only be as good as the therapists who deliver them. Many studies have noted wide variability across therapists in both patient retention and outcome in the treatment of AOD use disorders (Najavits and Weiss 1994). For example, Miller and colleagues (1980) found that among nine paraprofessional therapists treating alcoholics, success rates ranged from 25 to 100 percent. Moreover, the researchers found that therapist effects accounted for more than one-half of the variance in drinking outcomes for up to 1 year following treatment.

Training participating psychotherapists is an important strategy to reduce variability in treatment implementation in clinical studies. However, Morley and colleagues (1996) found that only 10 percent of the studies they reviewed provided such training. In clinical studies, training of experienced clinicians usually entails seminars that briefly review the range of interventions that will be delivered during the study. Following this didactic training, therapists usually complete at least one closely supervised training case. This experience provides the therapists with an opportunity to learn to adapt their usual approaches to protocol guidelines and to practice the techniques used in the trial (Carroll et al. 1994). These training cases are often conducted as part of a larger pilot study to determine the feasibility and efficiency of the study procedures before the study begins in earnest. Although the impact of training on therapist variability and skill level has not yet been demonstrated empirically, therapist training likely enhances the uniformity of treatment implementation in clinical trials (Crits-Christoph et al. 1991). Ongoing studies are evaluating whether manual-guided training of clinicians in community alcoholism treatment centers affects patient outcomes.

Treatment provider training is essential for both psychosocial and pharmacologic treatment studies. In pharmacologic trials, training is particularly important, because clinicians in these studies often face complex choices regarding such issues as the management of side effects and adverse reactions, patient dissatisfaction, and complications associated with comorbid disorders. These issues can lead to variability in treatment if some uniformity is not achieved through training (Carroll 1997*b*). Moreover, the National Institute of Mental Health’s Treatment of Depression Collaborative Research Project has demonstrated that even experienced pharmacotherapists benefit from training on delivering pharmacotherapy uniformly and skillfully. Pharmacotherapists in that study required more training and supervision than did psychotherapists to ensure competent and consistent delivery of therapy (Elkin et al. 1985).

#### Quality Control Procedures

To ensure that the treatment studied is implemented correctly, treatment delivery also must be monitored during a trial. For pharmacotherapies, this entails regularly assessing the patients’ compliance with the medication regimen. Commonly used assessment strategies include using medication vials with MEMS caps, monitoring riboflavin levels in the urine, and counting pills (Anton 1996). In some cases, more direct assessment procedures are performed, such as measuring the medication blood levels. Other approaches to enhancing compliance are to provide patients with feedback about their compliance throughout the trial and to ask them to self-monitor their compliance (e.g., through the use of diaries). Several quality control strategies also exist for monitoring the delivery of psychotherapy during a trial. For this purpose, treatment sessions usually are videotaped or audiotaped, and the tapes are subsequently reviewed by supervisors. These reviews typically focus on the degree to which the therapists deliver the treatment in adherence with manual guidelines and on the skill with which the treatment is delivered. Ongoing supervision throughout a clinical trial can have several benefits, including identifying and correcting changes in the therapist’s treatment delivery during a trial and maintaining therapist morale. Reviews of taped treatment sessions can be particularly useful for highlighting situations (i.e., therapeutic choice points) in which important clinical process issues arose and the therapist had to choose from several options. The analysis of such choice points provides supervisors and therapists with an opportunity to explore how the needs of particular patients can be met while adhering to a specific protocol. This information can help extend the therapists’ skills and repertoire and keep the treatment “fresh” throughout the trial. By using checklists or reviewing their own videotaped sessions, clinicians also can self-monitor their adherence to the study protocol (Carroll et al. in press).

#### Manipulation Checks

These measures, which are linked to quality control procedures, evaluate the extent to which a study treatment is successfully implemented and assess whether patients have adequate exposure to the treatment to which they are assigned. Manipulation checks for pharmacotherapies usually include such information as what the compliance rates with the study medication are and whether they differ among treatment conditions. For manipulation checks of psychotherapies, independent evaluators who are blind to the subjects’ treatment condition typically assess factors such as the degree to which therapists adhere to manual guidelines, deliver the treatment skillfully, and refrain from delivering interventions characteristic of comparison treatments. Manipulation checks (i.e., process measures) also can provide information on the extent to which a treatment’s theoretical “active ingredients” are present in the treatment (e.g., whether attendance at Alcoholics Anonymous meetings is higher in a 12-step–oriented treatment than in comparison approaches) and whether those ingredients are associated with better treatment outcomes.

### Strategies To Improve Outcome Assessment

In recent years, researchers have adopted several strategies for carefully characterizing study populations and their responses to treatment. One strategy involves the collection of appropriate and sufficient outcome data. The current consensus is that assessment instructions should fulfill the following requirements:

- They should be multidimensional; that is, investigators should collect data on a range of psychosocial functioning in addition to alcohol use.
- They should be multiperspective; that is, they should be based on reports from the patient, independent evaluators, and collateral informants (e.g., family members) as well as on biochemical markers.
- They should be longitudinal; that is, they should include regular followups for at least 1 year after treatment completion.
- Whenever possible, they should be obtained using standardized assessment instruments of established reliability and validity and with adequate sensitivity to changes.

In addition to these requirements, several methodological conventions specify how data should be collected. For example, data collection should be separated from treatment provision to reduce demand characteristics (i.e., the patients’ perception of pressure to provide positive reports of the study treatment). This can be achieved by using independent evaluators who do not know to which the treatments the subjects were assigned to and by assuring the patients that the collected data will not be shared with members of the treatment team. Data collection also should include all patients randomized to treatment, regardless of whether they drop out of treatment, because outcome assessment only of the select group of patients who comply with or complete treatment can introduce several sources of bias and undermine the validity of the results (Lavori 1992).

Because information on subjects’ drinking to date still relies heavily on the subjects’ self-reports, these data must be collected in an environment that enhances the accuracy of self-reports. Procedures that promote the accuracy of self-reports include explicitly assuring patients of confidentiality, minimizing negative consequences of reporting alcohol use, assessing subjects’ sobriety during assessment appointments, and using collateral reports or biochemical measures to confirm the patients’ drinking status (Babor et al. 1987). Several promising, sensitive biological markers of drinking are now available, including carbohydrate-deficient transferrin (CDT) (Anton 1996). CDT is a compound produced by the liver that is generated in higher quantities during periods of heavy alcohol consumption. Its concentration in the blood can be determined and is a sensitive and specific marker of heavy drinking. CDT may have several advantages over more traditional indicators of heavy drinking, such as gamma-glutamyl transferase.

## Linking Clinical Research and Clinical Practice

Although the ultimate purpose of clinical treatment research is to improve clinical practice, these two fields have, unfortunately, developed along separate lines. Traditionally, the most widely practiced alcoholism treatments have had little research-based support, whereas well-supported treatments have been used comparatively rarely in clinical practice (Miller et al. 1995). Currently, however, demands by managed care organizations and other pressures are increasingly compelling providers to demonstrate the effectiveness and value of the services they deliver and to provide treatments of demonstrated effectiveness. These pressures may help generate important new bridges between clinical research and clinical practice, including the adoption in clinical settings of methods that previously were reserved for research settings.

It is likely that most of the “cutting-edge” research methods described in this article will eventually be applied in clinical settings. For example, managed care reimbursement guidelines dictate that patients meet the criteria for recognizable, treatable syndromes. This requirement may lead to more widespread use of standardized diagnostic criteria, such as those described in the DSM and ICD systems, in clinical settings. Similarly, insurance approval for longer, more intensive treatment of patients with complex diagnoses may require confirmation of psychiatric comorbidity and concurrent psychosocial problems and, thus, the wider use in clinical settings of valid diagnostic instruments, such as the SCID, the DIS, and the Addiction Severity Index. Clinicians and treatment programs also will be called upon to demonstrate the effectiveness of the treatments they deliver. Accordingly, these providers will have to document the clinical outcomes of their patients on an ongoing basis, thereby leading to a more widespread adoption of standardized outcome instruments and of multidimensional assessment procedures. Furthermore, providers may be required to establish the durability of treatment and its value. As a result, long-term followup to assess outcome as well as cost offsets (i.e., reductions in relapse rates or in the usage of costly medical services) may become more common. Finally, because AUD treatment is associated with high rates of attrition and poor medication compliance, ongoing compliance monitoring also may become part of standard care in “real-world” clinical settings. This may include the use of MEMS caps and other strategies, such as compliance contracts,^3^ to encourage compliance with pharmacotherapies.

Manual-guided treatments with demonstrated effectiveness are already being adopted more frequently by clinicians. Because merely reading a manual does not confer expertise, however, more widespread use of manual-guided treatments will require more training and supervision of the clinicians who deliver these treatments. Moreover, treatment programs may wish to implement quality control procedures, including process assessment, that could be useful in evaluating the competence with which therapists administer treatments and their ability to foster good working relationships with their patients. With some manual-guided treatments, their adoption in clinical settings also may lead to more widespread familiarity with and use of standardized assessment instruments. For example, a treatment called motivational enhancement therapy (Miller et al. 1992), which is an effective brief treatment for problem drinking and AUD’s (Holder et al. 1991), involves objective feedback to the patient, based on an assessment of the patient’s level of drinking and consequences.

The adoption of other methodologies associated with clinical research also may improve outcomes in clinical settings. For example, the training and supervision of therapists, which is highly variable among clinical settings, itself may significantly improve treatments and their effectiveness by providing therapists with an opportunity to monitor and reflect on their work and to incorporate the observations and suggestions of supervisors. Taping of treatment sessions for review by supervisors also might help clinicians focus as well as encourage them to do their best work. However, although ongoing supervision of therapists and close clinical monitoring may likely contribute to comparatively good outcomes in clinical research studies (e.g., through Hawthorne effects^4^ and other mechanisms), this hypothesis has yet to be proven empirically.

Bridging the gap between clinical research and clinical practice will present new challenges not only to clinicians but also to researchers. Thus, researchers must take responsibility for the transfer of effective new treatments and methodologies to clinical settings. This transfer process can involve offering training in the use of validated treatments and psychometrically sound outcome assessments, making treatment and research manuals available, and evaluating research questions of particular interest to clinicians. Moreover, researchers should develop and evaluate treatments geared to the wide variety of AUD patients seen in clinical settings. More research is needed on whether the adoption of strategies typically associated with clinical research (e.g., use of manuals, provider training, compliance monitoring, and quality control procedures) in clinical settings actually improves outcomes. By collaborating with one another and informing each other’s work and progress, researchers and clinicians will be better able to meet the patients’ needs and the coming challenges posed by managed care.

## Footnotes

1 Throughout this article, the terms “efficacy” and “effectiveness” both are used. These two terms differ slightly in meaning. Efficacy refers to the results obtained under ideal treatment conditions, such as those present during rigorous clinical trials, whereas effectiveness refers to the results obtained under normal conditions of treatment delivery. Researchers generally first examine the efficacy of a treatment in a controlled trial and then conduct effectiveness studies to determine whether similar effects occur under “real-world” treatment conditions.

2 Acamprosate is an experimental drug that has not yet been approved for use in the United States.

3 Compliance contracts are agreements between the patient and another person (e.g., spouse) that encourage the patient to comply with therapy. For example, the patient and his or her spouse could agree that the spouse watches the patient take the medication once a day.

4 A “Hawthorne effect” is the finding that simply monitoring a behavior can change that behavior.
